# Intaglio Surface Adaptation of Removable Partial Denture Framework Fabricated by Various Data Acquisition Techniques and Fabrication Approaches

**DOI:** 10.1055/s-0043-1772245

**Published:** 2023-09-20

**Authors:** Seehachart Limpiwatana, Noppavan Nagaviroj

**Affiliations:** 1 Residency Training in Prosthodontics, Department of Prosthodontics, Faculty of Dentistry, Mahidol University, Bangkok, Thailand; 2 Department of Prosthodontics, Faculty of Dentistry, Mahidol University, Bangkok, Thailand

**Keywords:** CAD/CAM, accuracy, adaptation, 3D printing, removable partial denture

## Abstract

**Objectives** The aim of this study was to compare intaglio surface adaptation of the removable partial denture framework among various data acquisition techniques and fabrication approaches using three-dimensional comparison by metrology software.

**Materials and Methods** The partial edentulous typodont model with five digital superimposition landmarks was duplicated and scanned for the digital reference model. Three approaches were the conventional lost-wax (group I; LWT, *n* = 5), intraoral digital impressions combined with PolyJet printing and lost-wax (group II; IP-LWT, *n* = 5), and extraoral digital impressions combined with PolyJet printing and lost-wax (group III; EP-LWT, *n* = 5). Each framework was scanned and superimposed with the reference model. The misfits at 53 locations were measured.

**Statistical Analysis** Data were statistically analyzed by one-way analysis of variance, followed by Tukey's honestly significant difference for pairwise comparisons ( *p* < 0.05).

**Results** Significant differences were found between three approaches at the reciprocal arm, terminal part of the retentive arm, rest, and major connector ( *p* < 0.05). In the LWT group, the reciprocal arm and palatal vault region of major connector had the lowest misfits, but the highest misfit was found in the midline region ( *p* < 0.001). In the IP-LWT group revealed the most excessive contact at the terminal part of the retentive arm (-0.111 ± 0.038 mm, *p* = 0.031), with the highest misfit at the rest area ( *p* < 0.001).

**Conclusion** A difference in adaptation was found in several removable partial denture framework components among three approaches. The LWT group had a better adaptation than other groups. Nevertheless, a clinically acceptable adaptation was seen in all three approaches.

## Introduction

Removable partial dentures (RPDs) have been considered one of the treatment options in partially edentulous patients. However, the poor adaptation of the RPD is one of the common postinsertion problems. In addition, the risk of gingival inflammation is doubled if the RPD does not fit properly. 1 The conventional lost-wax technique (LWT) for fabricating the RPD involves numerous processes and depends on the lab technician's competence. Errors and inaccuracies can happen during impression taking, cast pouring, mounting on the articulator, cast duplication, waxing and investing, casting and finishing metal frameworks, flasking, and acrylic resin processing. 2 3 4 5 The errors are accumulated in each step, so the more procedures are involved, the more errors and failures could occur in prostheses. 2 3 4

Digital impression by the intraoral scanner is reported to have more advantages than conventional impressions by reducing the time-consuming steps (tray selection, dispensing and setting of material, disinfection, and shipment of the impressions to the laboratory), eliminating the problems from conventional impression materials (improper tray selection, distortion while taking impressions and pouring gypsum, and dimensional change of impression and construction of the working model), and enhancing patient's comfort. Furthermore, in terms of accuracy, intraoral scanner digital impressions are reported to be as accurate as conventional impressions 6 and clinically acceptable when used for RPD fabrication. 7 However, scanning errors could happen during the process of “stitching” the data, which is the process of putting together several smaller scans. 8 Any axis can have errors, but more errors were found in the Z-axis, necessitating a more careful scan in areas with different depths, such as areas with partial edentulousness or deep cavities. 9 Furthermore, some considerable numbers of dentists do not own the intraoral scanner. The reasons may include the dentist's expertise, technology adaptation, practice size, and financial constraints. 10

Computer-aided design/computer-aided manufacturing (CAD/CAM) fabrication techniques such as subtractive and additive manufacturing have been used for RPD fabrication. The subtractive manufacturing process removes material from a raw block to create an object of the desired size and shape. 11 12 In contrast, additive manufacturing is laying down successive layers of material to create an object. Many additive manufacturing methods have been used in RPD fabrication, 12 13 such as stereolithography, digital light processing (DLP), PolyJet, and selective laser melting. Additive manufacturing is more popular than subtractive manufacturing because it produces less waste and can create complicated or small structures than the size of the milling burs. 11 13 PolyJet or the DLP-jetting technique 13 is the method of extruding the photopolymer material in each layer and instantly curing the photopolymer with ultraviolet light. The PolyJet method is reported to have the lowest discrepancy among other three-dimensional (3D)-printing techniques. 14 15 It is commonly utilized for dental and medical applications, including surgical templates for implant placement, 16 the sacrificial pattern of RPDs, 17 18 and bioactive scaffolds. 19

For digital RPD framework fabrication, the CAD software is used to survey the undercut and design the framework after the data acquisition process. Then, the completed design data is sent to the 3D printing machine creating a sacrificial pattern. The sacrificial pattern is then directly cast into cobalt-chromium (Co-Cr) metal without a refractory model. 20 21 Due to the decreased number of manufacturing processes and human participation, this method might result in fewer cumulative mistakes in RPD production. 20 21

Various methods have been proposed for measuring the adaptation of frameworks, including indirect measurement using impression materials, 17 22 23 24 visual evaluation with a microscope, 25 and a micro-computed tomographic (CT) imaging system. 18 Due to the complexity and variety of RPD constructions, these techniques can only evaluate specific locations. Therefore, they do not reflect the overall adaptation of the RPD architecture. The digital superimposition method matches and compares two standard tessellation language (STL) files by metrology software. It has been proposed in various studies 6 and claimed to have a registration error of only 0.000224 ± 0.000071 mm in the full arch model. 26 This approach may also color map the prosthesis' overall adaptation and assess the distance between the framework and master model in various locations at once. However, few researchers apply the digital superimposition approach to compare the adaptation of RPD frameworks. 27 28

This study aims to compare intaglio surface adaptation of RPD framework by three approaches: (1) conventional LWT, (2) intraoral digital impressions combined with PolyJet printing and lost-wax technique (IP-LWT), and (3) extraoral digital impressions combined with PolyJet printing and lost-wax technique (EP-LWT) using 3D comparison by metrology software. The null hypothesis was that there was no statistically significant difference in intaglio surface adaptation of RPD framework among three approaches.

## Materials and Methods

**Fig. 1:**
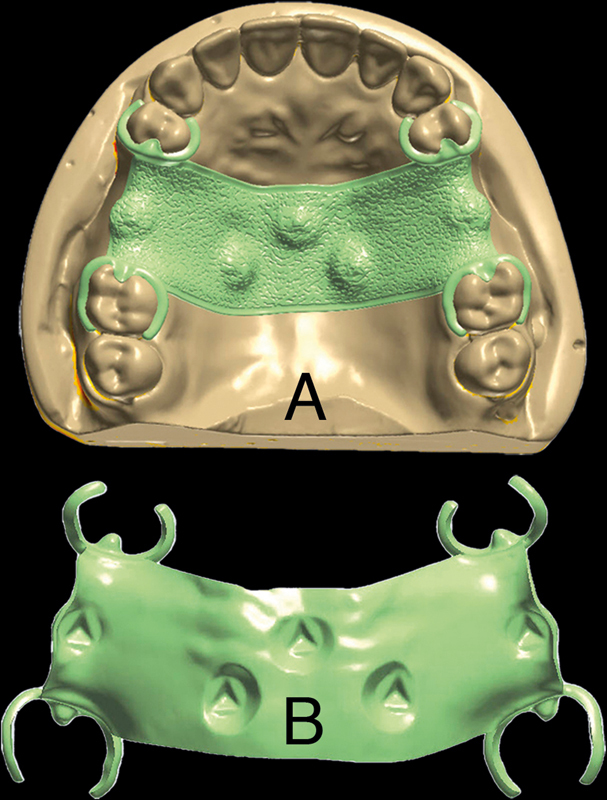
Design of the removable partial denture framework. ( **A** ) Overall design on the representative sample model. ( **B** ) Intaglio surface of the framework.

**Fig. 2:**
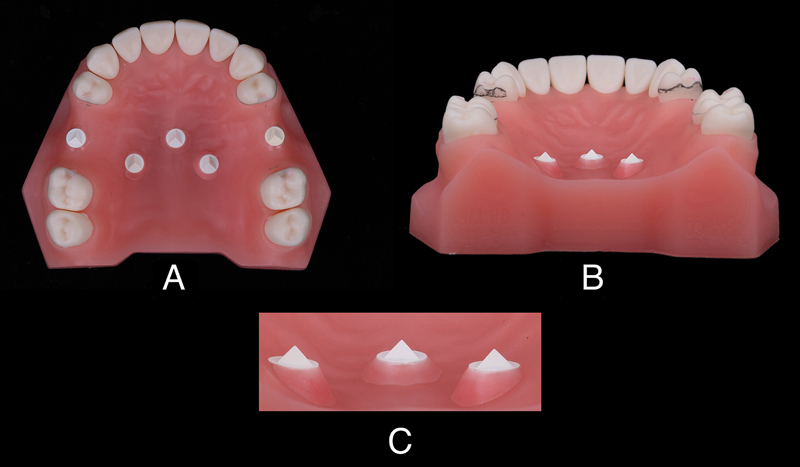
The prepared preliminary model. ( **A** ) Teeth preparation for removable partial denture and five digital superimposition landmarks fabricated with Cerec Bluecam scanbodies. ( **B** ) and ( **C** ) Undercut around the scanbodies were blocked out with modeling pink wax.

The preliminary model was the partially edentulous maxilla typodont model with the second premolars and first molars missing (A-3 TOK 136 model, Practicon Inc., North Carolina, United States). The RPD design included distal occlusal rests and Akers clasps engaged at 0.25 mm mesiobuccal undercut of the premolars (tooth 14, 24), mesial occlusal rests, and Akers clasps engaged at 0.25 mm distobuccal undercut of the molars (tooth 17, 27), reciprocal arms on palatal aspects of these abutments, and a palatal strap major connector ( Fig. 1 ). The landmarks for the digital superimposition of the model and the metal framework were created by inserting five scanbodies of Cerec Bluecam (Dentsply Sirona, North Carolina, United States) into the typodont model ( Fig. 2 ). The positions of the scanbodies were established using a dental surveyor and a tripoding technique. At first, the preliminary model was attached to the cast holder. Then, the analyzing rod was lowered from the surveyor's spindle, and the model was moved until three points at the same height touched the model's palatal tissue. Then, the surveyor's spindle was moved to the edentulous areas (15–16 and 25–26 areas) to find the other two points. Five circles were drawn with a carbon pencil. These circles are the location of Cerec Bluecam's scanbodies. The spaces and undercuts around the scanbodies were blocked out with modeling pink wax (Cavex Holland BV, Haarlem, the Netherlands) to form a tapered dome ( Fig. 2 ). The preliminary model was replicated and poured with epoxy resin EP-089 (Concrete Composite, Bangkok, Thailand). This new epoxy resin model, referred to as the reference model, was used throughout the study to fabricate metal framework specimens. The reference model was scanned with a Ceramill Map600 laboratory scanner (Amann Girrbach AG, Koblach, Austria). An STL file was generated to serve as the reference data set for all groups.

Fifteen experimental models and metal frameworks were fabricated in three groups. The sample size was determined by G*power software 29 (version 3.1.9.4; Heinrich-Heine-Universität Düsseldorf, Düsseldorf, Germany) by an a priori analysis with the effect size *f* = 1.2797168, *α* = 0.05, and 1-β = 0.95. The first group is the conventional lost-wax technique (group I; LWT); the reference model was duplicated five times by Polyvinyl siloxane (PVS) impression (Honigum Mono, DMG Chemisch-Pharmazeutische Fabrik GmbH, Hamburg, Germany) by the custom tray that provided uniform impression thickness for 3 mm 30 in all areas to minimize the influences of impression materials. The impression was poured with dental stone type 4 (M-Dent Dental Gypsum Dental Stone Type 4, Noritake SCG Plaster Co., Ltd., Bangkok, Thailand) using a vacuum mixing machine. The master casts were then given a survey undercut, block out, and relief (Ney Parallometer System, Dentsply Sirona, Pennsylvania, United States). The master casts were then duplicated into refractory casts and positioned the sacrificial patterns (Casting Wax, BEGO GmbH & Co. KG, Bremen, Germany). They were then invested (VR investment, Dentsply Sirona, Pennsylvania, United States) and cast into metal frameworks (Vitallium Alloy, Dentsply Sirona, Pennsylvania, United States).

The second group is the intraoral digital impressions combined with PolyJet printing and lost-wax technique (group II; IP-LWT); five scans were performed on the reference model using an intraoral scanner (3Shape TRIOS 3D Intraoral Scanner, 3Shape A/S, Copenhagen, Denmark). Between each scan, the scanner was turned off and restarted to simulate the individual data acquisition between samples. The STL data were exported and designed in the 3Shape Dental System CAD software (3Shape A/S, Copenhagen, Denmark). Five sacrificial patterns were printed using a 3D printer (Objet260 Dental 3D, Stratasys, Minnesota, USA) and MED610 PolyJet photopolymer (Stratasys, Minnesota, United States). Later, the sacrificial patterns were invested (CAD-Vest, Nobilium, New York, United States) and cast into five metal frameworks (Vitallium Alloy, Dentsply Sirona, Pennsylvania, United States).

The third group is the extraoral digital impressions combined with PolyJet printing and lost-wax technique (group III; EP-LWT); five surveyed master cast models without undercut block out and relief from group I were scanned with Ceramill Map600 laboratory scanner. The STL data were exported and designed on CAD software. Then five sacrificial patterns were printed by a 3D printer (Objet260 Dental 3D, Stratasys, Minnesota, United States) with MED610 PolyJet photopolymer (Stratasys, Minnesota, United States). After that, the sacrificial patterns were invested (CAD-Vest, Nobilium, New York, United States) and cast into five metal frameworks (Vitallium Alloy, Dentsply Sirona, Pennsylvania, United States).

After the casting process, all of the metal frameworks were roughly finished. The sprue of the casting was not removed. Instead, the sprue was used to function as the post to retain frameworks inside the plasticine when scanned with the laboratory scanner. In addition, no polishing was made on the intaglio surface to minimize human error from the manufacturing process and increase the study's validity. This methodology is based on a study by Brudvik and Reimers, 31 who reported an average of 0.127 mm of metal loss from the surface after finishing and polishing Co-Cr frameworks. All metal framework intaglio surfaces were scanned using a Ceramill Map600 laboratory scanner.

The STL file of each metal framework was superimposed with the reference model by a metrology software, Geomagic Control X 2020 (3D Systems, South Carolina, United States). The polished surface of the framework was removed by the selection and polygon settings tools to prevent software misrecognition during digital superimposition. After the polished surface was removed, the intaglio surface was inverted to make the five scanbodies reference marker on the reference model and metal framework coordinate in the same z-axis so the program may apply point-to-point matching and best-fit alignment appropriately. The sampling rate was set at 100%, with a maximum iteration count of 30. After best-fit alignment was done, the 3D compared command was created. The color surface mapping was created with a color bar range at ± 0.31 mm and a tolerance bar (green color) at ± 0.05 mm. A comparison point command was created with the “pick points” method, and the radius of the point to be measured was set at 1 mm.

**Fig. 3:**
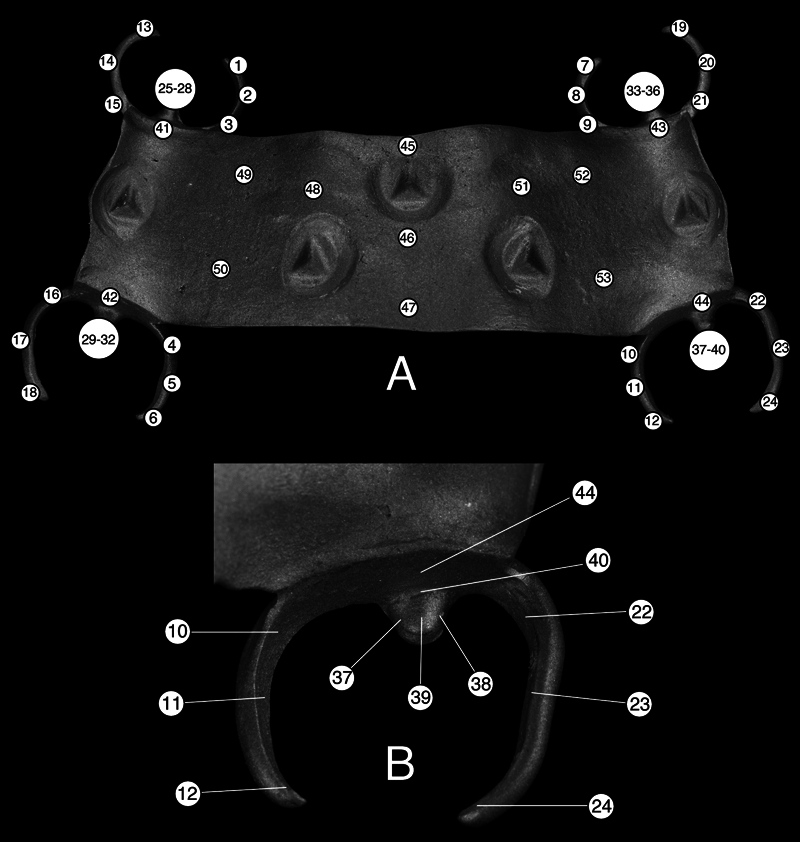
( **A** ) Measurement points throughout the framework; Points 1–12: shoulder, middle, and terminal end of the reciprocal arm. Points 13–24: shoulder, middle, and terminal end of the retentive arm. Points 25–40: center zone, marginal zone, and peripheral zone of occlusal rest. Points 41–44: proximal plate area. Points 45–53: major connector area (Points 45–47: midline area, Points: 48–53: palatal vault area). ( **B** ) Measurement points enlarged on clasp assembly of tooth 26. Point 10: shoulder of the reciprocal arm. Point 11: middle of the reciprocal arm. Point 12: terminal end of the reciprocal arm. Point 22: shoulder of the retentive arm. Point 23: middle of the retentive arm. Point 24: terminal end of the retentive arm. Point 37–38: peripheral zone of occlusal rest. Point 39: center zone of occlusal rest. Point 40: marginal zone of occlusal rest. Point 44: proximal plate.

Intaglio surface adaptation of RPD framework was assessed by the mean discrepancy between the reference model and 53 measurement points on the RPD framework; measurement points 1-12: shoulder, middle and terminal end of the reciprocal arm, measurement points 13-24: shoulder, middle and terminal end of the retentive arm, measurement points 25 to 40: center zone, marginal zone, and peripheral zone of occlusal rests, measurement points 41 to 44: proximal plate area, and measurement points 45-53: major connector area ( Fig. 3 ). The exact measurement points were accurately recorded in the coordinate of 3 axes (x, y, and z) and saved as the measurement template. The same template was used for all experimental groups; therefore, all measurement points were identical. The distance between the RPD frameworks and the reference model was recorded in millimeters.

For statistical analysis, IBM SPSS Statistics for Windows version 26.0 (IBM Corp., New York, United States) was used. The Shapiro–Wilk test and Levene's test were performed to validate the normality of the data and homogeneity of variance. A one-way analysis of variance was used to compare the mean discrepancy of the reference model and RPD framework components between all groups. A multiple comparison test, Tukey's honestly significant difference post hoc was used to determine the pair differences of each approach. Statistical significance was determined at *p-* value less than 0.05.

## Results

**Fig. 4:**
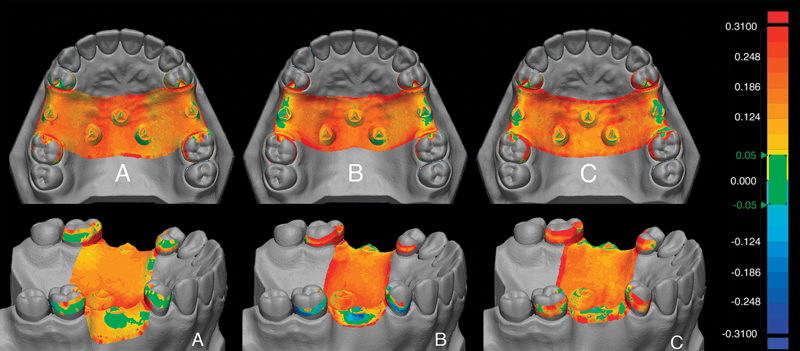
Color mapping of representative samples in occlusal and lateral view. ( **A** ) group I (lost-wax technique). ( **B** ) group II (intraoral digital impressions combined with PolyJet printing and lost-wax). ( **C** ) group III (extraoral digital impressions combined with PolyJet printing and lost-wax.).

The distance between the reference model and each framework was calculated in millimeters (mm). Fig. 4 depicts the color mapping of surface matching differences between all experimental groups. Green areas represent the ideal adaptation of the frameworks. In contrast, red areas suggest a gap between the reference model and the metal frameworks, and blue areas represent areas of pressure or compression between the metal frameworks and the reference cast. The framework adaptation from 0 to 0.05 mm was deemed close contact (no gap), 22 32 and the distance between 0.05 and 0.31 mm was deemed clinically acceptable. 21 27 28 After analyzing a total of 53 measurement areas, there was a statistically significant difference between the reciprocal arm, terminal part of the retentive arm, rest area, and major connector components ( *p* < 0.05, Table 1 ).

**Table 1:** The overall mean discrepancy between the reference model and frameworks in each component between all groups

Components	Specific components	Group I Mean (mm) ± SD	Group II Mean (mm) ± SD	Group III Mean (mm) ± SD	*p* -Value
Reciprocal arm	Shoulder	0.081 ± 0.010 a	0.216 ± 0.018 b	0.211 ± 0.018 b	<0.001 *
	Middle	0.054 ± 0.012 a	0.205 ± 0.018 b	0.219 ± 0.016 b	<0.001 *
	Terminal	0.034 ± 0.012 a	0.153 ± 0.018 b	0.179 ± 0.014 b	<0.001 *
Retentive arm	Shoulder	0.020 ± 0.013	0.046 ± 0.014	0.036 ± 0.020	0.541
	Middle	0.022 ± 0.013	0.019 ± 0.031	0.072 ± 0.039	0.406
	Terminal	−0.037 ± 0.028 ^a, b^	-0.111 ± 0.038 a	0.058 ± 0.048 b	0.031 *
Rest	Center	0.110 ± 0.015 b	0.228 ± 0.006 a	0.118 ± 0.022 b	<0.001 *
	Marginal	0.052 ± 0.013 b	0.125 ± 0.015 a	0.014 ± 0.007 b	<0.001 *
	Peripheral	0.071 ± 0.009 a	0.170 ± 0.010 b	0.124 ± 0.014 c	<0.001 *
Proximal plate	–	0.161 ± 0.020	0.171 ± 0.017	0.192 ± 0.022	0.535
Major connector	Midline areas (3 areas)	0.213 ± 0.010 a	0.150 ± 0.006 b	0.152 ± 0.009 b	<0.001 *
	Palatal vault areas (6 areas)	0.152 ± 0.003 a	0.207 ± 0.004 b	0.202 ± 0.004 b	<0.001 *

Abbreviation: SD, standard deviation.

* Group I (LWT), conventional lost-wax; Group II (IP-LWT), intraoral digital impressions combined with PolyJet printing and lost-wax; Group III (EP-LWT), extraoral digital impressions combined with PolyJet printing and lost-wax.

a,b,c Mean pairs with different superscripts represent the statistical difference ( *p* <0.05).

The mean discrepancy at the shoulder, middle, and terminal parts of the reciprocal arm were the lowest in group I (0.081 ± 0.010 mm, 0.054 ± 0.012 mm, and 0.034 ± 0.012 mm, respectively; *p* < 0.001, Table 1 ). The color mapping also displayed more green areas than groups II and III ( Fig. 4 ). At the region of the retentive arm, there were no statistically significant differences between groups at the shoulder and middle part. However, at the terminal part of the retentive arm, group III had the highest misfit (0.058 ± 0.048 mm), and group II had the most excessive contact (−0.111 ± 0.038 mm; *p* = 0.031, Table 1 ). The excessive contact of group II was shown in dark blue areas. In contrast, the highest misfit of group III was shown in red areas ( Fig. 4 ). Group II had the highest misfit at the center and marginal zones (0.228 ± 0.006 mm and 0.125 ± 0.015 mm, respectively; *p* < 0.001, Table 1 ). At the peripheral zone, group II also had the highest mean discrepancy (0.170 ± 0.010 mm), whereas group I had the lowest mean discrepancy (0.071 ± 0.009 mm). The differences between all three groups were statistically significant at the peripheral zone ( *p* < 0.001, Table 1 ). The misfit at the marginal zone was the lowest when compared to other zones.

Group I had the smallest gap at the proximal plate area (0.161 ± 0.020 mm). However, there were no statistically significant differences between groups ( *p* > 0.05, Table 1 ). At the midline area of the major connector, group I has the highest mean discrepancy (0.213 ± 0.010 mm) ( *p* < 0.001, Table 1 ). However, in the palatal vault area comprising all the remaining six measurement locations, group I had the smallest mean discrepancy (0.152 ± 0.003 mm; *p* < 0.001, Table 1 ).

## Discussion

**Fig. 5:**
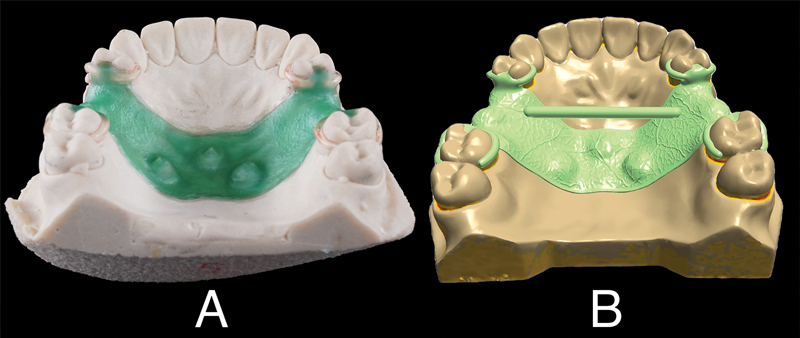
Different designs between the conventional lost-wax technique and three-dimensional printed sacrificial pattern. ( **A** ) The refractory cast with the wax pattern, absence of the support bar. ( **B** ) The computer-aided design that has to be incorporated with the extra support bar before printing.

This study aims to compare the intaglio surface adaptation of RPD frameworks fabricated by three approaches. The null hypothesis was rejected due to statistically significant differences between groups at several RPD components. A similar discrepancy existed at the region of the reciprocal arm and the palatal vault regions of the major connector. The LWT group had a significantly lower mean discrepancy than the IP-LWT and EP-LWT groups. The similarity between the IP-LWT and EP-LWT groups was the sacrificial pattern fabrication technique. The MED610 photopolymer was used to fabricate the sacrificial pattern, which has been reported to have a 0.1 mm geometric discrepancy. 33 Errors in additive manufacturing can also be found in complex designs, such as those with a deep curvature and a steep slope, which was at the major connector area. In addition, the 3D printed sacrificial pattern in this study was created with the horizontal support bar on the Y-axis on the major connector area connecting from the left to right direction. The purpose of the support bar was to prevent distortion during the printing and investing procedure. However, it was not necessary for the LWT group ( Fig. 5 ). During the casting procedure, the localized casting shrinkage at the point where the support bar meets the major connector may happen, 34 35 which can cause misfit at both of the reciprocal arm and the palatal vault region of the major connector. This finding suggests that more studies are required to determine the effect of the support bar on RPD framework accuracy.

On the contrary, the LWT group had the highest misfit at the midline area of the major connector. Diwan et al 5 discovered that the prefabricated wax pattern for RPD has a stress relaxation effect, 36 causing the wax pattern to straighten out and attempt to return to its original shape. As a result, the wax sheet could lift off the cast at the center of the palatal area, potentially causing the RPD framework to misfit. However, the 3D printed resin pattern did not have the stress relaxation effect because it was designed and manufactured to the desired shape. Hence, the LWT group had more misfits in the midline area of the major connector than the IP-LWT and EP-LWT groups. To reduce major connector shrinkage and mismatch, according to the literature, adding the reservoir on each sprue might constantly supply the molten alloy during the solidification shrinkage of the framework, resulting in fewer gaps and enhancing casting quality in the major connector area. 37

The mean discrepancy at the retentive arm region was comparable to other studies, 27 38 which were less than 0.31 mm. 21 and the terminal region of the retentive arm revealed excessive contact, which was recorded as negative numbers. 27 28 Moreover, the mean discrepancy at the terminal part of the retentive arm had the least misfits compared to the middle and shoulder parts. 39

The marginal zone at the rest area displayed the lowest mean discrepancy than the center and peripheral zones. These findings may be attributed to the thicker metal in the marginal zone, which causes less shrinkage and distortion. 22 Moreover, the peripheral zone had a closer adaptation than the center zone, which is consistent with the findings of earlier studies. 22 24 Furthermore, the rest seat's marginal zone must be beveled, while other areas were butted or horizontal. The beveled edge may influence better seating. 40 The most significant mean discrepancy was found at the rest seat in the IP-LWT group. This discrepancy may be caused by the negative figures at the terminal portion of the retentive arm, which could hinder the seating of the rest components. 23

The inaccuracies observed in the IP-LWT and EP-LWT groups may be caused by the removal of the supporting structure from the 3D printing process, which had to be done before the investing procedure, thereby increasing the risk of distortion. In addition, the dimensional stability of the sacrificial pattern and handling temperature may also contribute to the misfits in the RPD framework. Other variables that could cause the inaccuracy are the scanning strategy, the process of transforming the scan data into a 3D model, and the filter algorithms of the scanner. 6 9

In this research, the intaglio surface adaptation of the RPD framework was evaluated using the digital superimposition approach, which removes the drawbacks of previous techniques. For instance, the elasticity of impression materials may affect the measuring procedure and resulting in inaccuracies. 24 The metal artifact between the micro-CT and Co-Cr framework restricts measurement areas to specific sites. 18 Furthermore, the teeth and RPD frameworks must be altered to have an atypical appearance, such as the buccal ledge on the abutment teeth for the placement of the retentive clasp and the nonengaging undercut retentive clasp, in order to measure the gap using light microscopy. 25 The digital superimposition approach can evaluate the adaptation in all components of the RPD frameworks at once, having the exact location in every measurement and only 0.0002 mm registration errors. 26 Moreover, the teeth or RPD components can be designed in a conventional manner, such as the retentive clasp that engages in the undercut area, thereby enhancing the study's validity.

Finishing and polishing the intaglio surface of the RPD framework may affect the adaptation of the prosthesis. Moreover, other RPD designs and various 3D printing procedures and materials, such as selective laser melting, may result in different outcomes; therefore, these factors should be explored in future research.

## Conclusion

A difference in intaglio surface adaptation was found in several RPD framework components among three approaches. The LWT group had a better adaptation when compared to other groups. On the other hand, the IP-LWT group had the poorest adaptation at the rest area. Nevertheless, a clinically acceptable adaptation was seen in all three approaches.
